# Promising Chemotherapy for Malignant Pediatric Brain Tumor in Recent Biological Insights

**DOI:** 10.3390/molecules27092685

**Published:** 2022-04-21

**Authors:** Qian Zhou, Yichen Xu, Yan Zhou, Jincheng Wang

**Affiliations:** 1 Department of Pharmacy, Hangzhou Medical College, Hangzhou 310053, China; qianz1220@hmc.edu.cn (Q.Z.); 13777892445@163.com (Y.Z.); 2 Department of Biological Sciences, University of Southern California (Main Campus), Los Angeles, CA 90007, USA; yichenx@usc.edu; 3 Center for Drug Safety Evaluation and Research, Zhejiang Province Key Laboratory of Anti-Cancer Drug Research, College of Pharmaceutical Sciences, Zhejiang University, Hangzhou 310058, China

**Keywords:** brain tumor, histone deacetylase, tyrosine kinase, traditional chemotherapy, novel agents

## Abstract

Brain tumors are the most widespread malignancies in children around the world. Chemotherapy plays a critical role in the treatment of these tumors. Although the current chemotherapy process has a remarkable outcome for a certain subtype of brain tumor, improving patient survival is still a major challenge. Further intensive treatment with conventional non-specific chemotherapy could cause additional adverse reactions without significant advancement in survival. Recently, patient derived brain tumor, xenograft, and whole genome analysis using deep sequencing technology has made a significant contribution to our understanding of cancer treatment. This realization has changed the focus to new agents, targeting the molecular pathways that are critical to tumor survival or proliferation. Thus, many novel drugs targeting epigenetic regulators or tyrosine kinase have been developed. These selective drugs may have less toxicity in normal cells and are expected to be more effective than non-specific chemotherapeutics. This review will summarize the latest novel targets and corresponding candidate drugs, which are promising chemotherapy for brain tumors according to the biological insights.

## 1. Introduction

Brain tumors have become the most prevalent solid neoplasm in children under the age of 14 in recent years, with an average yearly age-adjusted incidence rate of 23.79 per 100,000 people in the United States [1]. The incidence of pediatric brain tumors has gradually elevated by 18.32% in 2013–2017 compared with 2009–2013 [2], highlighting the need for the precise and early diagnosis of a pediatric malignant brain tumor [3].

Despite the improvements in diagnosis, brain tumors are still the predominant cause of death in childhood. Among all patients, malignant brain tumors have an even worse prognosis. A combination of neurosurgery, chemotherapy and/or radiotherapy is necessary, while many survivors suffer from obvious chronic neuroendocrine and neurocognitive impairments [4,5,6,7]. To increase the efficacy of cancer treatment while lowering its toxicity, systematic chemotherapy is carried out and has been confirmed to be very helpful. Moreover, chemotherapeutic scientists have extensively evaluated the treatment of pediatric brain tumors, including molecular insights [8], epigenetics [9] and the genome targeted landscape [10].

Malignant pediatric brain tumors (PBT) are classified into distinct subtypes by the cell types and locations, including glioblastoma and diffuse intrinsic pontine glioma (DIPG), which are both derived from glial cells and medulloblastoma contributed by neuron cells. This review highlights the promising chemotherapy method for the treatment of pediatric brain tumors using different molecular targets, and also briefly discusses their molecular pathological epidemiology.

## 2. Histone Deacetylase Inhibitors

Histone acetylation is one of the epigenetic modifications that occur without causing a change in the core DNA sequence [11]. The balancing function between acetyltransferases (HATs) and histone deacetylases (HDACs) is crucial to regulating gene expression in human cancers [12]. HDACs are highly expressed in many cancers, so the HDAC inhibitors are a powerful new class of small-molecule therapeutics in many cancers, including pediatric brain tumors [13].

### 2.1. Panobinostat

Panobinostat is a multi-HDAC inhibitor [14], in 2015, Grasso, C.S., et al. conducted a chemical screening in DIPG cultures taken from patients and identified that panobinostat was one of the most effective agents [15]: among 16 DIPG cultured cells, 12 of them are demonstrated sensitivity to panobinostat, leading to the decreased expression of proliferation-related genes Mki67 and Ccnd1. Furthermore, they observed an obvious inhibitory effect on orthotopic xenograft tumors. This inhibitor has also been studied in pre-clinical experiments by using the genetic BSG mouse model and the H3.3K27M orthotopic DIPG xenograft model, which confirmed the inhibitor’s efficiency as an antitumor agent [16,17]. In another experiment, panobinostat enhanced the gene therapeutic effect of hAT-MSC.sTRAIL [18], decreasing tumor growth and prolonging the survival rate [19]. The combined treatment of panobinostat and other agents, such as AXL inhibitor BGB324, bromodomain inhibitor JQ1, or the CDK7 inhibitor THZ1, also resulted in synergistic antitumor effects on DIPG cells [20,21]. This inhibitor has undergone clinical trial I against DIPG and is now recruiting participants in children.

Studies show that panobinostat may exhibit poor blood-brain barrier (BBB) penetration [22,23]. Umberto Tosi et al. [24] designed a PET-visible analog to the HDAC inhibitor panobinostat to address this issue, named PETobinostat. PETobinostat has the advantages of convection-enhanced delivery (CED) and an image-guided visible delivery system, which allow for monitoring and optimal efficacy. These studies suggest that this could be a promising DIPG therapy.

### 2.2. Valproic Acid

Valproic acid (VA) is a fatty acid with 8-carbon branched-chains. It has been shown to be beneficial in the treatment of seizure disorders and to be an inhibitor of histone deacetylase (HDAC) [25]. More than a decade ago, valproic acid was studied for the treatment of pediatric malignant glioma [26,27]. XN Li reported that it could inhibit the growth of medulloblastoma cell lines, correlated with a reduction in TP53, CDK4, and CMYC expression and the activation of p21 [28]. Another study showed that valproic acid could increase histone H3 acetylation, indicating HDAC inhibition, and inhibit DIPG cell viability through the promotion of apoptosis [29]. Valproic acid could reduce the proliferation of glioma stem cells and invasion by regulating Wnt/β-catenin signaling [30]. It was also reported that valproic acid enhanced the anti-glioma effect of temozolomide through the p53–PUMA apoptosis pathway [31]. A phase I study of valproic acid in children with CNS malignancies was recently conducted by the Children’s Oncology Group [32]. Patients tolerated valproic acid three times daily to maintain trough concentrations of 75–100 μg/mL, according to the researchers. At this dose, no dose-limiting toxicities were identified, which suggests a high degree of safety. One patient with DIPG (glioblastoma) had a validated partial response, while another patient (DIPG) had a small reaction, indicating the potential anti-glioma effect of VA. Recently, a nationwide population-based cohort analysis in Taiwan showed that VPA could improve OS in HGGs TMZ treatment with a total database of 2379 patients [33].

Valproic acid followed by maintenance bevacizumab has been investigated in children with recently diagnosed high-grade gliomas or brainstem gliomas in a phase two study of valproic acid and radiation, indicating valproic acid did not appear to increase the EFS or OS of children with DIPG when combined with bevacizumab, although they were well tolerated [34]. Therefore, valproic acid or its combination with other antitumor drugs needs to be further studied as a potential promising candidate therapy for pediatric brain tumors.

### 2.3. Vorinostat (SAHA)

The Food and Drug Administration (FDA) has approved Vorinostat as the first HDAC inhibitor for the chemotherapy of cutaneous T-cell lymphoma [35]. Before this, Ilker Y. Eyüpoglu demonstrated that SAHA had potential anti-glioma properties in vivo, ex vivo and in vitro by up-regulating cell cycle protein p21/WAF, as well as introducing apoptosis [36]. To date, few clinical trials have been conducted. Vorinostat has been shown to be beneficial as a treatment for pediatric HGG in a trial conducted by the Children’s Oncology Group [37,38]. However, it is worth noting that a complete response was recorded in a patient with neuroblastoma, and one of the seven patients with DIPG was observed to have a prolonged stable disease. Other phase I/II studies for diagnosed glioblastoma and recurrent solid tumors are also underway. SAHA has been combined with other signaling pathway inhibitors, including temozolomide and bortezomib. Although no objective response has been observed to date, hopefully, further progress will be published in the near future.

### 2.4. Others

Similar to SAHA, Trichostatin A (TSA) is a pan-HDAC inhibitor that slows the proliferation of various cancer cells. Several studies show that TSA inhibits glioma cell development by inducing cell cycle arrest and death in a P53-dependent manner, confirming its anti-tumor impact on glioma cells [39,40].

Sodium butyrate (NaB) could inhibit cell survival in human MB cell lines and might also promote the neuronal differentiation of MB cells since the compound suppresses the growth or survival of cancer stem cells [41,42].

In vitro and in xenografts, Corin, a bifunctional inhibitor of HDACs, and LSD1 effectively reduce DIPG growth. Corin raises H3K27me3 levels, which are repressed by H3K27M histones, while simultaneously increasing HDAC-targeted H3K27ac and LSD1-targeted H3K4me1 in differentiation genes [43]. Another drug that targets both HDAC and PI3K elicits a robust cytotoxic effect in pHGG and DIPG models by inhibiting NFB and FOXM1-mediated DNA damage and could also elevate radiosensitive in DIPG [44]. MPT0B291 is a new HDAC inhibitor that has been shown to diminish cell viability and promote cell damage in human and rat glioblastoma cell cultures, but not in healthy astrocytes, partially through the acetylation of p53 [45]. HDAC6 is also a potential target for glioma. Studies showed that Tubastatin A [46], Ricolinostat [47] and JOC1 [48] could significantly reduce tumorigenesis both in vitro and in vivo.

**Figure 1:**
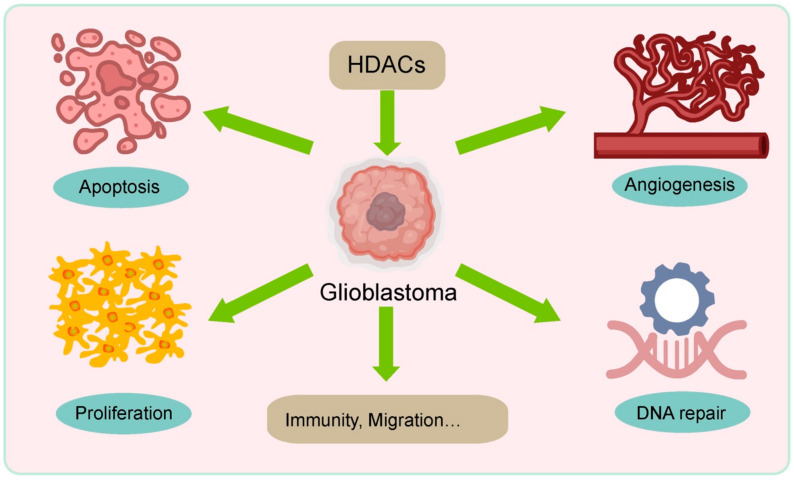
Role of HDACs in Glioblastoma.

Above all, HDAC inhibitors have multiple effects on the treatment of tumor cells including apoptosis, blocking the cell cycle, and inhibiting DNA repair and so on (Figure 1), which makes them a promising target for the treatment of pediatric brain tumors. An increasing number of HDAC inhibitors, such as romidepsin [49], NK-HDAC-1 [50] and Belinostat [51] are being studied in preclinical or clinical research, and we are eager to receive breaking news regarding the treatment of pediatric brain tumors.

## 3. Tyrosine Kinase Inhibitors

Most growth factor receptors contain a tyrosine kinase sequence, called the TK receptor, comprising epidermal growth factor receptor (EGFR), platelet-derived growth factor receptor (PDGFR), and vascular endothelial growth factor receptor (VEGFR), etc. [52]. The activation of TK controls the activity of many targets, such as Ras/MAPK, STAT, JNK, and PI3K, in cells. For example, phosphorylation of the TK receptors combines with downstream targets and induces the mitogen-activated protein kinase (MAPK) and PI3K-AKT signal pathway, which are critical for cell division and survival. Therefore, the activation of TK leads to cell proliferation and disables apoptosis, both of which are directly related to tumor initiation and formation. Hence, TK inhibitors can potentially kill tumor cells, and many TKIs have great potential as a form of tumor treatment [53].

### 3.1. Sunitinib

Sunitinib (SU11248, Sutent) is a multi-target oral molecule that inhibits several tyrosine kinases related to tumor angiogenesis, including VEGFR, PDGFR, c-KIT, FLT3 and RET. The FDA has approved it for the treatment of renal cell carcinoma (RCC) and imatinib-resistant gastrointestinal stromal tumor (GIST) [54]. Sunitinib has been reported to inhibit medulloblastoma tumor cells by inhibiting the STAT3 and AKT signaling pathways [55]. Unfortunately, no patients showed a response rate in a clinical trial II studying for high-grade glioma [56].

### 3.2. Dasatinib

Dasatinib is an anti-cancer drug, which inhibits Bcr-Abl tyrosine kinase, and has been authorized for chronic myelogenous leukemia (CML) as a first-line treatment. More excitingly, Dasatinib can pass the blood-brain barrier and be used as a treatment for Philadelphia chromosome–positive leukemia in the central nervous system [57], which demonstrates the drug’s potential to defend against pediatric brain tumors. Recently, Sagar Agarwal revealed that dasatinib can go through the BBB and inhibit glioma growth [58]. Another study by Miriam Benezra used the novel fluorinated dasatinib derivative (F-SKI249380) combined with a nanocarrier vehicle to treat the high-grade glioma in a mouse model and found a positive inhibitory efficacy [59]. In addition to these studies, dasatinib has been used in a clinical trial I with another VEGFR/EGFR inhibitor vandetanib [60] for the treatment of DIPG. The penetrability of Dasatinib makes it very promising that this kind of therapy has a bright future.

### 3.3. Crenolanib

Crenolanib is a second-generation FLT3 inhibitor that is now being tested in clinical studies for its safety and efficacy in the treatment of several cancers, including acute myeloid leukemia (AML) and gastrointestinal stromal tumors (GIST). It can mainly inhibit both wild type and mutant FLT3 at very low concentrations, as well as PDGFRa, which will increase the growth inhibitory effect, especially in peritumor tissue-derived cancer stem cells (p-CSC) [61]. Recently, one clinical trial I study has been completed in DIPG, but the results have not been published to date [62].

### 3.4. Recombinant Humanized Monoclonal Antibody

The use of monoclonal antibodies (mAbs) to treat cancer has shown to be one of the most effective treatments for solid tumors in the last two decades. Strictly speaking, this is a form of immunotherapy, since mAb can target malignant cells using several mechanisms, such as VEGF, EGFR, IGFR and HER2 [63]. The most-studied mAb is anti-VEGF mAb Bevacizumab—six clinical trials are being, or have been, conducted, with four of those being related to malignant brain tumors. It is a little disappointing that Bevacizumab seems to be more efficient in low-grade gliomas than those of a high grade [64]. Bevacizumab has been shown to reduce mortality and morbidity in recurrent GBM in adult patients when used in combination therapy (most frequently irinotecan). Based on the positive results of two promising phase 2 studies, the FDA granted bevacizumab accelerated approval as an agent for recurrent GBM. It seems that Bevacizumab’s activity depends on the age of the patients [65]. However, clinical trials in pediatric high-grade glioma are still ongoing. Another clinical trial shows that, in pediatric DIPG patients, Nimotuzumab treatment was well-tolerated, and there was modest nimotuzumab activity in DIPG, which means that a small population of DIPG patients appear to benefit from anti-EGFR antibody treatment [66]. The same condition applies to anti-EGFR mAb Cetuximab, for which clinical trial II in HGG/DIPG is ongoing, followed by radiation therapy.

The other TKIs and mAb chemotherapies include the VEGF inhibitor pazopanib, the VEGFR inhibitor cediranib, c-Met inhibitor tivantinib, and the ALK/ROS1 inhibitor crizotinib, the mTOR inhibitor temsirolimus and the IGFR mAb cixutumumab, MEK1/2 inhibitor Trametinib [67]. These targeted chemical medicines could specifically inhibit the brain tumor region, which brings more hope for treatment in childhood. A summary of histone deacetylase inhibitors and tyrosine kinase inhibitors in clinical trials and the preclinical stage are listed in Table 1.

**Table 1:** Typical clinical trials and preclinical agents of histone deacetylase inhibitors and tyrosine kinase inhibitors.

Agents	Clinical Trial	Phase	Disease Model	Refs
Panobinostat	NCT04341311	I	Diffuse intrinsic pontine glioma (DIPG)	[20,21]
Valproic acid	NCT00107458	I/II	Brain and Central Nervous System Tumors	[32,33,34]
Vorinostat	NCT03426891	I/II	Glioblastoma/Brain Tumor	[37,38]
Trichostatin A	Pre-clinical	N/A	Glioblastoma	[39,40]
Sodium butyrate	Pre-clinical	N/A	Medulloblastoma	[41,42]
Corin	Pre-clinical	N/A	Diffuse intrinsic pontine glioma (DIPG)	[43]
MPT0B291	Pre-clinical	N/A	Glioblastoma	[45]
Tubastatin A	Pre-clinical	N/A	Glioblastoma	[46]
Ricolinostat	Pre-clinical	N/A	Glioblastoma	[47]
JOCI	Pre-clinical	N/A	Glioblastoma	[48]
Romidepsin	NCT00053963	I	Childhood High-grade Cerebral Astrocytoma	[49]
Belinostat	Pre-clinical	N/A	Glioblastoma	[51]
Sunitinib	NCT01462695	II	Glioblastoma/Brain Tumor	[56]
Dasatinib	NCT00788125	I/II	Brain and Central Nervous System Tumors	[60]
Crenolanib	NCT01393912	I	Diffuse intrinsic pontine glioma (DIPG)	[62]
Bevacizumab	NCT02157103	II	Glioblastoma	[65]
Nimotuzumab	NCT00753246	II	Glioblastoma Multiforme	[66]
Cetuximab	NCT01012609	II	Diffuse intrinsic pontine glioma (DIPG)	/
Pazopanib	NCT01931098	II	Glioblastoma Multiforme	/
Trametinib	NCT03434262	I	Glioblastoma	[67]

## 4. Traditional Chemotherapy

Although there are many novel compounds targeting brain tumors, there still are a couple of conventional chemotherapies approved by the FDA. Everolimus exhibited a sustained effect on subependymal giant-cell astrocytoma (SEGA), causing tumor reductions in a 5-year-old with tuberous sclerosis complex [68]. Lomustine is used in patients who have already undergone surgery or radiation therapy. Temozolomide, an alkylating and methylating drug, has demonstrated great response rates with reasonable tolerance in chemotherapy-naive patients with recurrent oligodendroglial malignancies [69] and has been combined with other compounds, such as bevacizumab, cilengitide, temsirolimus and SAHA, for use in clinical pediatric brain tumor. Furthermore, by inhibiting DHFR/TYMS, pemetrexed (PTX) showed synergistic anti-glioma action with TMZ in either glioblastoma cells or U251 xenografts [70]. Similarly, the selective inhibitors of CDK4/6, MicroRNA-29b, Methadone, ribavirin and other agents may enhance glioma cells’ sensitivity to TMZ [71,72,73,74,75]. In my opinion, although an increasing number of diverse inhibitors are being revealed, conventional medicines are still greatly valuable for specific brain tumor types and combination therapy.

## 5. Novel and Other Agents

In addition to the drugs or compounds mentioned above, there are many studies looking at chemotherapy treatments using either new molecules or new targeted inhibitors, which provide a new, promising trend in the treatment of pediatric brain tumors.

GSKJ4 (a selective jumonji H3K27 demethylase inhibitor) influences the proinflammatory macrophage response according to Rintaro Hashizume [76]. In K27M-expressing cells, an ethyl ester derivative of the H3K27 demethylase inhibitor increased K27me2 and K27me3, inhibited K27M glioma cell growth and prevented K27M colony formation in vitro, as well as demonstrating K27M anticancer efficacy in vivo. The authors proposed a treatment strategy for K27M pediatric gliomas that merits further exploration.

Similarly, this year, due to the up to 80% mutation rate for K27M in DIPG, Faizaan Mohammad also explored EZH2 inhibitors with tumor-suppressive functions [77]. He identified two small molecular compounds, GSK343 and EPZ6438, which abrogated tumor cell growth through the induction of the tumor-suppressor protein p16INK4A. This study provides a new target, EZH2, for the treatment of K27M-mutant pediatric gliomas.

BMI1, a component of the Polycomb repressive complex 1 (PRC1), could be a new target for DIPG treatment. According to Ilango Balakrishnan, inhibiting BMI1 reduces cell self-renewal and slows tumor growth via the induction of senescence. Long-term BMI1 suppression could cause a senescence-associated secretory phenotype and slow tumor growth. Inhibition of BMI1 would accelerate tumor cell death in vivo by clearing senescent cells [78].

Catherine S. Grasso [15] used chemical screening, RNAseq studies, and integrated computational models to uncover potentially viable therapy methods in patient-derived DIPG cells. They discovered that panobinostat is a promising DIPG treatment. Furthermore, panobinostat and GSKJ4 showed synergy in combination testing, implying that these findings could point to a promising DIPG therapy strategy.

In 2017, Surya Nagaraja [21] demonstrated that BRD4 or CDK7 inhibition can cause transcriptional dysregulation in DIPG, and BRD4 inhibitor JQ1 [79] and CDK7 inhibitor THZ1 [80] synergize with HDAC inhibition to target oncogenic transcription through either or both methods. Similarly, I-BET 151 [81], exhibited a promising anti-glioma effect by targeting BRDs. These findings revealed previously unknown DIPG mechanisms and pathobiology and provide new potential for DIPG treatment.

This year, Christin Schmidt [82] performed a preclinical drug screening for embryonic tumors with multilayered rosettes (ETMR), which is a rare but aggressive form of tumor in infants and young children. The study identified Topoisomerase inhibitors topotecan and doxorubicin, epigenetic agents decitabine and panobinostat, actinomycin D, PLK1 inhibitor volasertib, aurora kinase A inhibitor alisertib, and mTOR inhibitor MLN0128 were all identified as interesting candidates for future clinical trials. It is necessary to further explore these compounds and combinations.

Acid ceramidase has also been identified as a potential therapeutic target for adjuvant pediatric brain tumor therapy [83]. Carmofur has been used therapeutically in Japan for colorectal cancers since 1981, and it is a promising medicine that will be further studied in animals before being tested in humans as a treatment for pediatric children with brain tumors.

DIPG harbored somatic mutations in ACVR in a quarter of patients, encoding the serine/threonine kinase ALK2. A recent study demonstrated that pyridine LDN-214117 and pyrazolo[1,5-a] pyrimidine LDN-193189, could increase survival rate in orthotopic xenografts of H3.3K27M, ACVR mutant glioma cells, as ALK2 inhibitors. Further studies of ALK2 inhibitors will play an important role in the treatment of DIPG patients [84].

As we all know, the ubiquitin proteasome pathway regulates a variety of functions in the cell, and it’s also vital for tumor cell growth and survival [85]. Bortezomib is a very effective and selective proteasome inhibitor that has shown promising results in vitro and in vivo studies of a variety of solid and hematologic tumors [86]. Daniela A. Bota demonstrated that bortezomib and bevacizumab combination treatments improve efficacy. These medicines work in tandem to treat temozolomide-resistant malignant gliomas [87] because they have complementary mechanisms of action. In clinical trial II, GDC-0449 treatment resulted in rapid but transient tumor regression and symptom relief in a case with SHH medulloblastoma [17,88]. Another phase 3 trial comparing oral sonidegib (LDE225) to temozolomide (TMZ) in people with Hh pathway-activated relapsed medulloblastoma is now underway. These medicines provide targeted tumor types or individual treatment therapy.

## 6. Molecular Pathological Epidemiology in Pediatric Brain Tumor (PBT)

Although lots of promising chemotherapy treatments have been developed to improve the outcomes of pediatric brain tumor (PBT), an understanding of risk factors, such as parents, diet, and PBT environment is also critical, which may differentially influence molecular pathology and response to chemotherapy in each patient, and may increase pediatric cancer risk [89,90]. In this section, we briefly discuss the molecular pathological epidemiology research on PBT.

### 6.1. Reproductive Factor

The reproductive factor during pregnancy is an important risk factor for PBT. Medication may increase the risk of PBT. A study from Taiwanese showed that mothers taking herbs, such as Coptis increased their risk of PBT [91]. Moreover, mothers taking antihypertensive drugs showed a positive correlation with the occurrence of PBT in their offspring [92]. The use of other drugs, such as gastrointestinal drugs, analgesics, hormonal contraception and antiemetics, was not significantly associated with the risk of cancer in children [93,94].

Reproductive defects are a major cause of high-risk PBT. Studies have shown an increased risk correlated with high birth weight (>4000 g) [95,96]. Reproductive abnormalities are linked to a 2-fold increase in the incidence of PBT, according to large population-based research. PBTs were shown to be more common in children born with CNS birth disorders or neurological abnormalities [97].

Other reproductive factors, such as high gestational age, premature delivery, vaginal forceps and cesarean section also contribute to a high risk of PBT [98].

### 6.2. Environmental Factor

Environmental exposure is a critical element in PBT. Exposure to form moderate to high doses of ionizing radiation is the only identified environmental risk factor for PBT [99]. There is evidence that radiotherapy for early-onset childhood cancer could lead to an increased risk of brain tumors of up to 29% [100,101]. Similarly, maternal diagnostic radiation during pregnancy was associated with an increased risk of brain tumors in offspring. Non-ionizing radiation, including radiofrequency and microwaves, did not provide sufficient data to mark them as risk factors for PBT [99].

However, several studies supported the hypothesis that the residential use of pesticides, particularly insecticides during both pregnancy and childhood could increase the risk of PBT [102,103]. Thus, limiting the residential use of pesticides and monitoring the exposure might be necessary policies.

### 6.3. Dietary Factors

Studies have reported that some dietary factors affect the risk of PBT. A report showed a protective effect of prenatal vitamins/FA on offspring PBT risk, while dietary *N*-nitroso compounds were indicated to induce brain tumors in offspring [104]. Therefore, it may be meaningful to monitor the daily intake of these dietary factors to predict the possible risk of PBT in the clinic.

### 6.4. Germline Genetic Variations

Germline mutations linked to PBT differ by histologic subtype, with predisposition mutations found in roughly 10% of sporadic PBT cases. Recently, a major study using whole-exome sequencing on juvenile high-grade glioma discovered that the rare germline variations linked to the risk of PBTs are mostly found in twenty-four genes involved in DNA repair and cell-cycle pathways, primarily in the TP53 and NF1 genes [105]. Waszak and colleagues used rare variant burden analysis to estimate that germline mutations are responsible for 6% of medulloblastoma diagnoses and identified SUFU, PTCH1, APC, BRCA2, PALB2 and TP53 as consensus medulloblastoma risk genes [106]. The genetic variants of BRCA2 could be affected by environmental exposures, such as formaldehyde (i.e., gene-by-environment interactions) [107]. Begemann and colleagues studied 1044 medulloblastoma cases and discovered that heterozygous germline mutations in the GPR161 gene were solely related to the SHH subgroup, accounting for 5% of the SHH subgroup neonates in their medulloblastoma cohort [108]. Thus, these findings may identify some genes, such as TP53, NF1, SUFU, APC and PTCH1, as risk genes or clinical biomarkers for PBT.

## 7. Conclusions

Chemotherapy has played a critical role in the treatment of malignant brain tumors in children. Conventional chemotherapy elevates the survival rate regarding certain childhood brain tumors but is limited by its low specificity or resistance. Promising biological research has revealed specific targets and mutations in individual brain tumors, and developed novel targeted agents to achieve improved efficacy and low toxicity. The “one pill fits all” strategy will eventually be substituted by individualized treatment for specific brain cancers [109].

Although the future of tumor chemotherapy is undoubtedly exciting, there are still many deficits that need to be addressed. The presence of the blood-brain barrier(BBB), which prevents compounds from reaching the central nervous system (CNS), is a key cause of the tremendous difficulties in treating brain tumors. Accordingly, new drug delivery approaches, such as modifications to existing drugs, a nanosystem based on delivery, and the effective delivery of therapeutic peptides, are under development to combat the BBB. Another defect is the toxicity of the conventional and even targeted anti-tumor agents which is an unexpected outcome of chemotherapy for brain tumors. Special attention should be paid to the use of these agents in children. One of the new strategies for reducing toxicity is inhibiting a certain bacterial enzyme [110]. In the future, more approaches to minimizing the side effects of brain tumor medicine in childhood will be developed. Molecular pathological epidemiology research is a promising direction for the investigation of risk factors in relation to molecular pathologies and clinical outcomes.

In most cases, regular surgery and radiotherapy should be carried out for the treatment of malignant pediatric brain tumors. Based on conventional chemotherapy, novel molecular or targeted chemotherapy, or the combination of both treatments, could increase the possibility of curing these diseases, which suggests a more promising treatment avenue for those children.

## Data Availability

Not applicable.

## Sample Availability Statement

Not available.
